# Structural Design and Testing of a Micromechanical Resonant Accelerometer

**DOI:** 10.3390/mi13081271

**Published:** 2022-08-07

**Authors:** Heng Liu, Yu Zhang, Jiale Wu

**Affiliations:** School of Electronic & Information Engineering, Nanjing University of Information Science & Technology, Nanjing 210044, China

**Keywords:** accelerometer, resonant frequency, electrostatic stiffness, sensitivity

## Abstract

Micromechanical resonant accelerometers based on electrostatic stiffness have the advantage of it being possible to adjust their sensitivity by changing the detection voltage. However, there is a high-order nonlinear relationship between the output frequency and the induced acceleration, so it is difficult to obtain the theoretical basis to guide the microstructure design. In this study, the dynamic equation for this type of accelerometer was established under the condition of the stiffness of the folded beams being much less than that of the resonant beams. The sensitivity was obtained first, and then silicon-based microstructures were fabricated, for which metal tube-shell vacuum packaging was adopted. The two static driving capacitances were about 0.88 pF, and the detection capacitances were about 0.38 pF in the experimental test. The sensitivity was 44.5 Hz/g when the detection voltage was 1 V, while it was greater than 300 Hz/g when the detection voltage was 3 V. With an increase in the detection and driving voltages, a coupling phenomenon occurred between the vibration amplitude and frequency of the resonant beam. The double-stage folded beam failed at a high detection voltage larger than 10 V. Through the experiment, a numerical simulation model for the accelerometer was established, providing the basis for a closed-loop control circuit design.

## 1. Introduction

Electrostatic negative stiffness is widely used in the modal frequency matching of micro-machined gyroscopes and resonant accelerometers [1,2]. After conducting microstructure fabrication, designers can tune the sensitivity of an accelerometer through electrostatic negative stiffness [3]. The magnitude of the electrostatic negative stiffness is related to the parameters of plate capacitance and loading voltage [4]. Electrostatic stiffness resonant accelerometers mainly rely on two types of acceleration—the change in plate facing area [5,6] and plate distance [7,8]. The area change type is an out-of-plane twist of the microstructure beam, while the plate distance change type is an in-plane movement of the microstructure beam; the former is more nonlinear than the latter. When electrostatic negative stiffness is applied to accelerometer design, two movable micromechanical beams are required [9]: one is a resonant frequency-sensitive beam, and the other is a movable micromechanical beam that generates electrostatic negative stiffness. Both micromechanical beams are portable; therefore, calculating the negative electrostatic stiffness directly is difficult.

Due to the nonlinear relationship existing between the resonant frequency and the stiffness of the micromechanical beam [10], directly obtaining the sensitivity, which introduces complications in the structural design, is also difficult. As the electrostatic stiffness is generated by the DC voltage loaded on the plate capacitor, a change in acceleration will change the plate distance. An extremely large loading voltage will cause the plate to pull in and cause the microstructure beam to fail [11]. In the design stage, attention should be paid to the pull-in voltage of the plate [5]. The electrostatic stiffness resonant accelerometer can be used to realize the conversion of the loaded voltage into the electrostatic driving force through the interface capacitance [3,12]. Following driving and detection capacitance design is a necessary part of structural design [13]. Due to the parasitic capacitance between the microstructures [7], the driving electrode directly couples the driving voltage signal to the detection electrode, meaning that an open-loop test cannot be used to directly determine the resonant frequency. On the one hand, modulation and demodulation circuits need to be used; on the other hand, the parasitic capacitance needs to be reduced in the structural design.

This study introduces the principle of a resonant accelerometer based on electrostatic negative stiffness and proposes a structural design principle under the condition of stiffness constraints. Finite element simulation is used to validate the stiffness constraints, and the size of the interface detection capacitance and drive capacitance is obtained simultaneously. Static capacitance tests are conducted on the tape-out and packaged accelerometers, and a frequency sweep test is carried out with a dynamic signal analyzer to validate the effect of the DC voltage on the sensitivity. At the same time, the large detection voltage makes the deformation of the double-stage folded beam irreversible, and the large driving voltage used results in the coupling of the amplitude and the resonant frequency of the resonant beam. With the computer dimension measurement method, the numerical simulation model of the accelerometer is established, which provides the basis for the subsequent design and experimentation of the control circuit.

## 2. Principle of Accelerometer

**Figure 1:**
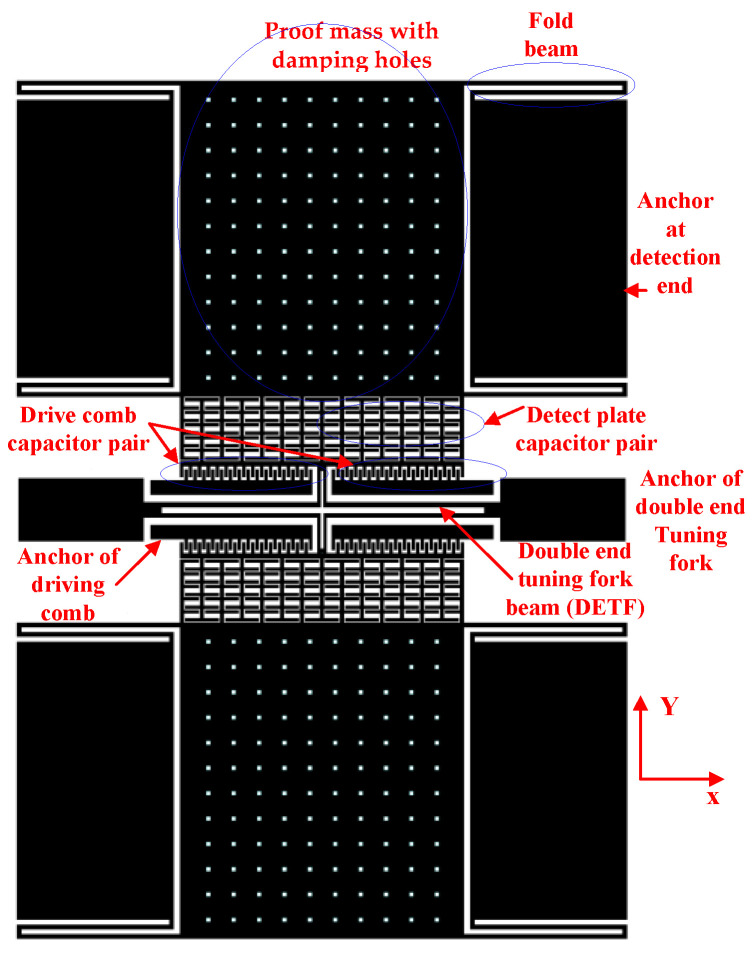
Structural diagram of an accelerometer.

The accelerometer structure layer is divided into two identical single-beam resonant accelerometers at the middle symmetry point in the Y-axis direction, as shown in Figure 1. Taking the upper part as an example, the structure layer includes a sensitive proof mass with some damping holes, four folded beams (single or double stage) supporting the suspended proof mass, a detection plate capacitor pair, two fixed-drive comb capacitor pairs, a tuning fork resonant beam, and some fixed anchors(two anchors at the detection end, two anchors for the driving comb, and two anchors for the double-end tuning fork). The Y-axis direction is the drive and detection direction.

**Figure 2:**
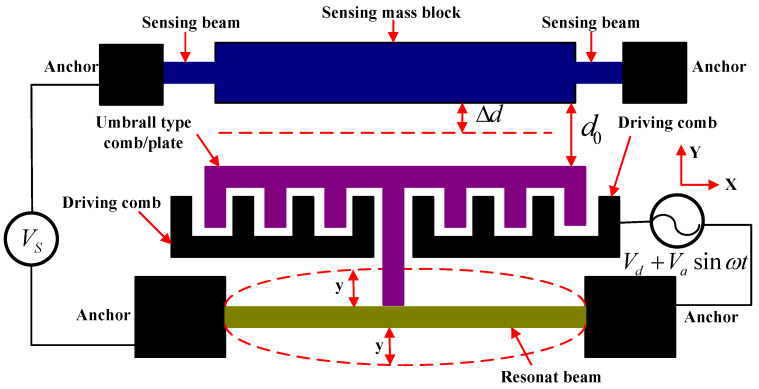
Equivalent diagram of an accelerometer.

The equivalent diagram of one accelerometer (Figure 2) shows that the folded beams are the sensing beams, and the two folded beams are a group. The tuning fork beam is the resonant beam. The flat plates attached to the mass and the flat plates attached to the tuning fork beam form detection capacitor pairs, and the driving combs attached to the tuning fork beam and the driving combs attached to the anchor form drive capacitor pairs.

Folded beams and proof-mass structural connect with detection voltage $V_{s}$. Both the driving combs and the active tuning fork beam link with a driving voltage $V_{d}+V_{a}\sin\omega t$. The tuning fork beam potential is 0. The dynamic equation of the resonant beam is [14]:

(1) $$m\ddot{y}\;+\;\xi \dot{y}+ky=F_{d}+F_{e}$$

In Formula (1), y is the modal displacement of the tuning fork beam, $F_{d}$ is the electrostatic driving force, k is the effective mechanical stiffness of the tuning fork beam, m is the equivalent mass, and the damping coefficient is ξ. $F_{e}$ is the electrostatic force of the detection plate capacitance acting on the resonant beam. The total capacitance of the detection plate is:

(2) $$C_{s}=\frac{\varepsilon Nhl}{d_{0}-y_{1}}=\frac{\varepsilon S}{d_{0}-y_{1}}$$

In Formula (2), N represents the pairs of the parallel plate capacitance, $d_{0}$ is the initial distance between the two plates of the detection capacitor, $y_{1}$ is the displacement of the folded beam in the Y-axis direction, ε is the dielectric constant, h is the overlapping thickness of the capacitor plates along the Z-axis direction, l is the overlap length of a single capacitor along the Y-axis direction, and S=Nhl is the equivalent area of the two plates of the detection capacitor.

The electrostatic force $F_{e}$ is

(3) $$F_{e}=\sum^{\;}\frac{1}{2}\frac{\partial C_{s}}{\partial y_{1}}V_{s}^{2}=\frac{N\varepsilon lhV_{s}^{2}}{2}\left(\frac{1}{d_{0}^{2}}+\frac{2y_{1}}{d_{0}^{3}}+o\left(y_{1}^{2}\right)\right)$$

The total capacitance of the drive comb $C_{d}$ is

(4) $$C_{d}=\frac{N_{0}\varepsilon h\left(l_{0}+y\right)}{d_{1}}$$

In Formula (4), $N_{0}$ represents the pairs of capacitances of the driving comb, $l_{0}$ is the overlapping length of combs in the Y-axis direction, $d_{1}$ is the distance between the comb and the next comb in the X-axis direction,$V_{d}$ is the DC driving voltage, and $V_{a}\sin\omega t$ is the AC driving voltage. The electrostatic driving force $F_{d}$ is

(5) $$F_{d}=\sum^{\;}\frac{1}{2}\frac{\partial C_{d}}{\partial y}{\left(V_{d}+V_{a}\sin\omega t\right)}^{2}=\frac{1}{2}\frac{N_{0}\varepsilon h}{d_{1}}{\left(V_{d}+V_{a}\sin\omega t\right)}^{2}$$

Substituting Equations (3) and (5) into Equation (1) and ignoring the higher-order terms of the electrostatic force, we have

(6) $$m\ddot{y}\;+\;\xi \dot{y}+ky=\frac{1}{2}=\frac{N_{0}\varepsilon h}{2d_{1}}{\left(V_{d}+V_{a}\sin\omega t\right)}^{2}+\frac{\varepsilon SV_{s}^{2}}{2d_{0}^{2}}$$

In Formula (6), the equivalent stiffness of the resonant beam $k_{eff}$ is

(7) $$k_{eff}=k-\frac{\varepsilon SV_{s}^{2}}{d_{0}^{3}}$$

According to Formula (7), under the detection of a plate loaded with $V_{s}$, the corresponding resonant frequency decreases when the equivalent stiffness of the resonant beam decreases, and the level of reduction is related to $V_{s}$ and $d_{0}$. The resonant accelerometer can be designed by establishing the relationship between the acceleration a in the Y-axis direction and the initial distance $d_{0}$.

According to Figure 2, the folded beams and the resonant beam were under an acceleration effect, which made the solution for the distance $d_{0}$ more complicated. The equivalent stiffness $k_{s}$ was much smaller than the modal stiffness k, and the detection proof mass $m_{s}$ was much larger than that of the resonant beam m. Under the action of the electrostatic driving force, the resonant beam performedhigh-frequency sinusoidal periodic vibration about the fixed equilibrium position, and the equivalent low-frequency displacement was 0.

When the acceleration in the Y-axis direction is 0, for the detection capacitance system,

(8) $$\frac{1}{2}\frac{\varepsilon SV_{s}^{2}}{{\left(d_{0}-\Delta d\right)}^{2}}=k_{s}\cdot \Delta d$$

When the acceleration in the Y-axis direction is not 0, the following holds true:

(9) $$\frac{1}{2}\frac{\varepsilon SV_{s}^{2}}{{\left(d_{0}-\left(y_{1}+\Delta d\right)\right)}^{2}}=k_{s}\left(y_{1}+\Delta d\right)-m_{s}\cdot a$$

In Equations (8) and (9), Δd and $y_{1}$ are the displacements of the folded beam and the proof mass in the Y-axis direction when the acceleration is 0 and not 0, respectively. The actual design should consider the pull-in effect of the plate capacitor; that is, the value $d_{0}$ should be as large as possible, but if it is too large, the detection of the output signal will become difficult. Generally, it will satisfy $y_{1}+\Delta d\ll d_{0}$. After expansion with the Taylor series, the relationship between displacement $y_{1}+\Delta d$ and acceleration a can be obtained as follows:

(10) $$y_{1}+\Delta d=\frac{m_{s}\cdot a}{k_{s}-\frac{\varepsilon SV_{s}^{2}}{d_{0}^{3}}}+\frac{\frac{\varepsilon SV_{s}^{2}}{2d_{0}^{2}}}{k_{s}-\frac{\varepsilon SV_{s}^{2}}{d_{0}^{3}}}$$

The resonant frequency $f_{e}$ of the resonant beam is:

(11) $$f_{e}=\frac{1}{2\pi }\sqrt{\frac{k-\frac{\varepsilon SV_{s}^{2}}{{\left(d_{0}-y_{1}\right)}^{3}{\left(1-\frac{-\Delta d}{d_{0}-y_{1}}\right)}^{3}}}{m}}$$

where

(12) $$\Delta d=\frac{\frac{\varepsilon SV_{s}^{2}}{2d_{0}^{2}}}{k_{s}-\frac{\varepsilon SV_{s}^{2}}{d_{0}^{3}}}\;,\;y_{1}=\frac{m_{s}\cdot a}{k_{s}-\frac{\varepsilon SV_{s}^{2}}{d_{0}^{3}}}$$

Sensitivity η is expressed as

(13) $$\left|\eta \right|\approx \frac{\delta f_{e}}{\delta a}\approx \frac{3\varepsilon SV_{s}^{2}/2\pi }{\sqrt{\left(kd_{0}^{4}-\varepsilon SV_{s}^{2}d_{0}\right)\cdot m}}\frac{m_{s}}{2k_{s}d_{0}^{2}-3\varepsilon SV_{s}^{2}/d_{0}}$$

where the frequency is related to the structural parameters and the detection voltage. The sensitivity can be improved by adjusting the loading voltage $V_{s}$, but the sensitivity is nonlinear with $V_{s}$. The greater the stiffness of the folded beam $k_{s}$ is, the smaller the sensitivity will be. It is necessary to configure the parameters reasonably in the structural design. The preceding analysis was conducted under the condition $k_{s}\ll k$—that is, the influence of the tuning fork beam on the resonant frequency due to the electrostatic force and inertial force can be ignored.

## 3. Structural Design of Accelerometer

**Figure 3:**
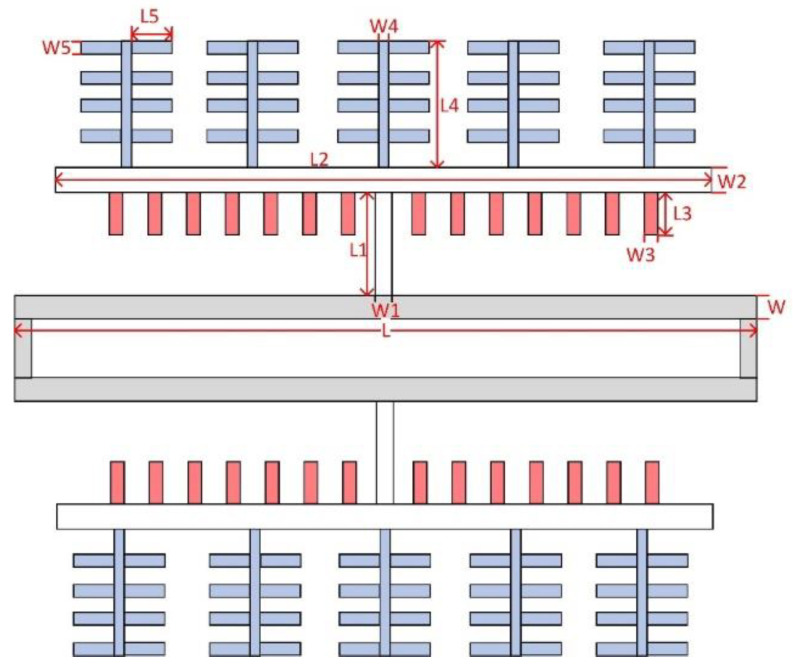
DETF resonator.

Theoretical analysis shows that when the stiffness of the folded beam is much lower than that of the resonant beam, the influence of the vibration displacement of the resonant beam on the detection capacitance can be ignored, and this constraint should be considered in the structural design. The tuning fork resonant beam and its connecting parts are shown in Figure 3. The dimensions of each key position are marked with symbols, and the stiffness and equivalent mass of the resonant beam corresponding to the operating mode can be calculated.

For a double-ended tuning fork (DETF) beam, the stiffness k and equivalent mass m can be expressed as

(14) $$k=\frac{256E\times W^{3}\times h}{15L^{3}}$$

(15) $$m=\frac{128}{315}\rho \times L\times W\times h+\rho \times A_{act}\times h$$

In Formula (15), the in-plane effective area of the additional plate $A_{act}$ is

(16) $$A_{act}=L_{1}\times W_{1}+L_{2}\times W_{2}+n_{1}\times L_{3}\times W_{3}+n_{2}\times \left(L_{4}\times W_{4}+n_{3}\times L_{5}\times W_{5}\right)$$

In Formulas (14)–(16), E is the Young’s modulus of silicon, ρ is the density, $A_{act}$ is the area of the additional structure, $n_{1}$ is the number of comb capacitors, $n_{2}$ represents the pairs of parallel plate capacitances, $n_{3}$ is the number of parallel plate capacitors, and other parameters are as shown in Figure 3.

**Figure 4:**
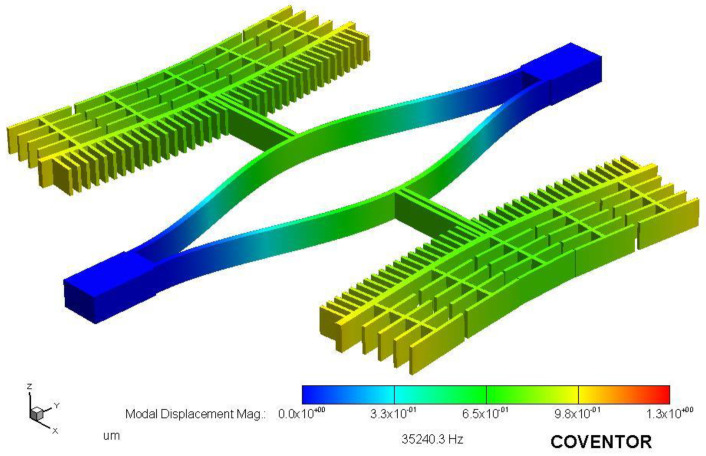
Finite element simulation of resonator.

In the simulation, attention was paid to the influence of the length L and width W of the tuning fork beam on the resonant frequency. Finite element simulation of the resonator structure was performed using CoventorWare software [15]. First, the 3D model was constructed according to the microstructure’s ideal process flow, and the resonant beam’s anchor points were constrained to be fixed, and then the modal simulation of the resonator structure was carried out. The first-order in-plane mode of the resonant structure is shown in Figure 4. When the length of the tuning fork beam was 700 μm and the width was 8 μm, the theoretically calculated resonant frequency was 38,296 Hz, the simulated frequency was 35,136.1 Hz, and the error was 8.99%. The stiffness of the resonance beam obtained from the simulation was 153.04 N/m and the theoretically calculated stiffness was 163.0439 N/m.

**Figure 5:**
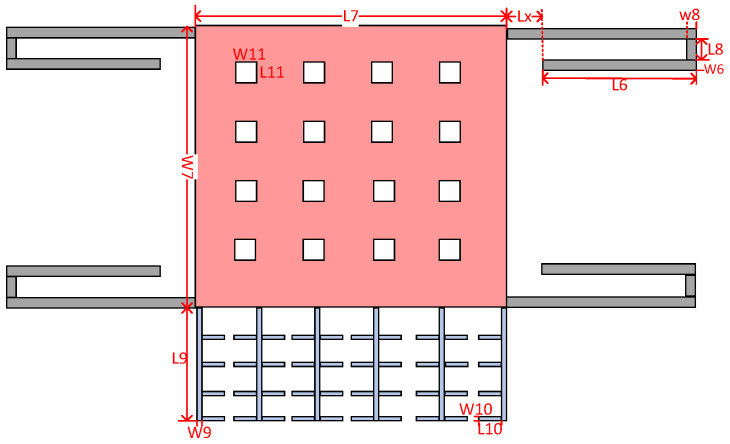
Finite element simulation of sensing structure.

The detection proof mass system consisted of a sensitive proof mass with damping holes, four folded beams (single-or double-stage), and additional parallel plates. The dimension symbols are shown in Figure 5.

When the straight beam $L_{6}$ is much longer than the connecting beams $L_{8}$ and $L_{X}$, ignoring the influence of the connecting beam, the values of stiffness corresponding to the single-stage $k_{s1}$ and double-stage folded beams $k_{s2}$ are

(17) $$k_{s1}=\frac{2\times E\times w_{6}^{3}\times h}{L_{6}^{3}}$$

(18) $$k_{s2}=\frac{E\times w_{6}^{3}\times h}{L_{6}^{3}}$$

**Figure 6:**
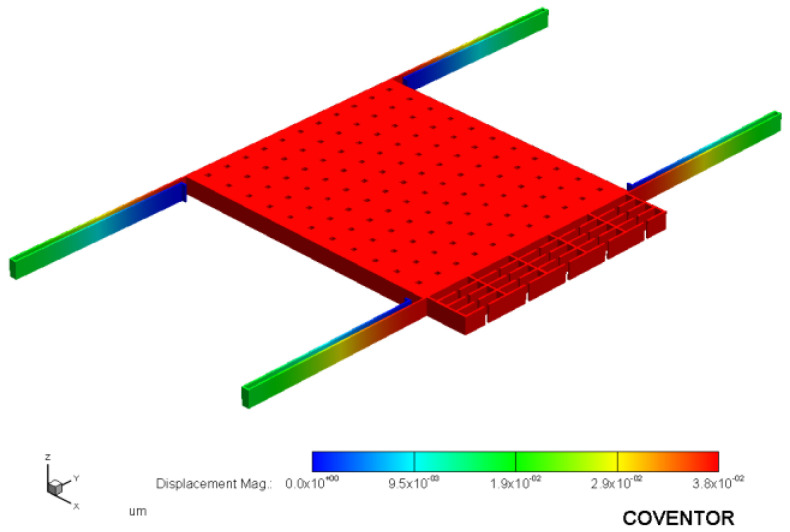
Static force analysis (single-stage beam).

**Figure 7:**
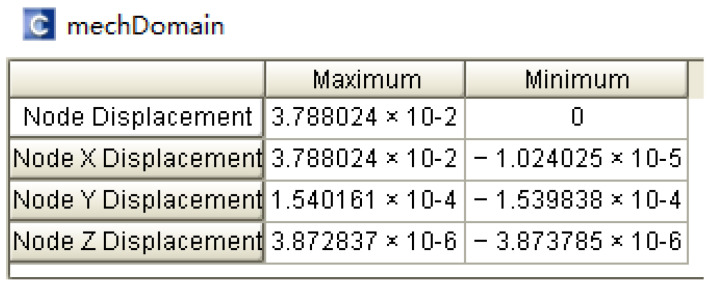
Displacement (um) of single-stage beam.

According to Equations (17) and (18), ignoring the influence of the connecting beams, when the length and width of the straight beams are the same, the stiffness of the double-stage folded beam is half that of the single-stage folded beam. When the length of the single beam is 445 μm and the width is 4 μm, the stiffness of the single-stage folded beam can be calculated to be 10.31 N/m, and that of the corresponding double-stage folded beam is 5.15 N/m, making the modal stiffness of the resonant beam k more than 15 times the stiffness of the single-stage folded beam $k_{s1}$ and the stiffness of the double-stage folded beam $k_{s2}$. After completing the 3D modeling of the microstructure in CoventorWare software, the force analysis of the sensing structure was performed to obtain the fold beam’s simulation stiffness, and the detection end anchor points were fixed, as shown in Figure 6. Different accelerations in the sensitive direction to simulate the force $F_{s}$ were applied, the displacement x in the sensitive direction was measured, and the expression $k_{s}=F_{s}/x$ obtained the simulation stiffness of the fold beam $k_{s}$, When the acceleration is 1 g, the simulation results are shown in Figure 7. The displacement results obtained after applying different accelerations in the X-axis direction (sensitive direction) are shown in Table 1 and Table 2. According to the simulation results, the stiffness of the folded beam under the experiment was9.4398 N/m and 5.1127 N/m, respectively. The relative errors from the theoretical stiffness were 8.44% and 0.71%, respectively.

The mass of the proof mass $m_{s}$ is calculated as follows:

(19) $$m_{s}=\rho \times A_{s}\times h$$

(20) $$A_{s}=4\left[L_{6}\times W_{6}+L_{8}\times W_{8}+\left(L_{6}+L_{x}\right)\times W_{6}\right]+L_{7}\times W_{7}-n\times L_{11}\times W_{11}+n_{4}\times L_{9}\times W_{9}+\left(n_{4}-1\right)\times n_{5}\times L_{10}\times W_{10}$$

**Figure 8:**
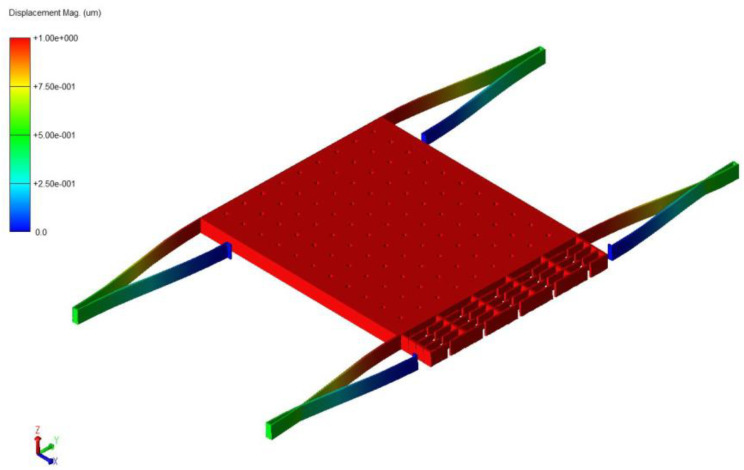
Finite element simulation of detection structure (single-stage beam).

**Figure 9:**
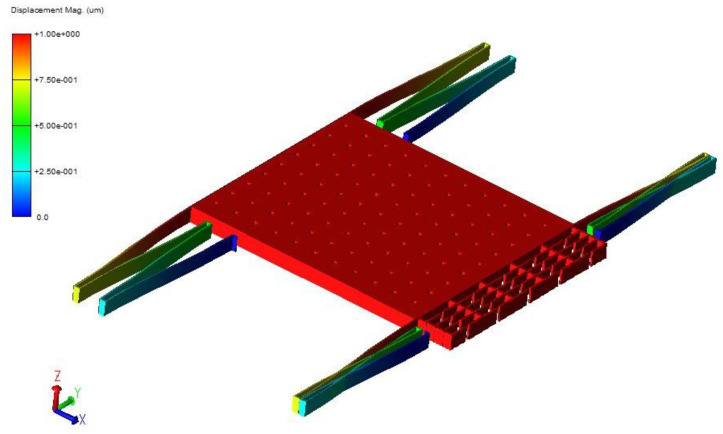
Finite element simulation of detection structure (double-stage beam).

To obtain the working mode and modal frequency of the sensing structure, we used the Coventor Ware software to model the single-stage/double-stage fold beam sensing structure in 3D first [16], and the corresponding anchor points were kept stationary, and then modal simulations on the sensing structure were performed, as shown in Figure 8 and Figure 9. According to the analysis, the operating modal frequency of the single-stage folded beam detection system was 2532.74 Hz, and the operating modal frequency of the double-stage folded beam detection system was 1864.01 Hz. The stiffness of the double-stage folded beam was confirmed to be 0.5416 of the stiffness of the single-stage folded beam. The modal frequency of the folded beam detection system was much smaller than the modal frequency of the resonant beam, meaning that the influence of the vibration of the resonant beam on the distance between the flat capacitor plates at the detection end can be ignored.

## 4. Manufacturing and Characterization Testing

**Figure 10:**
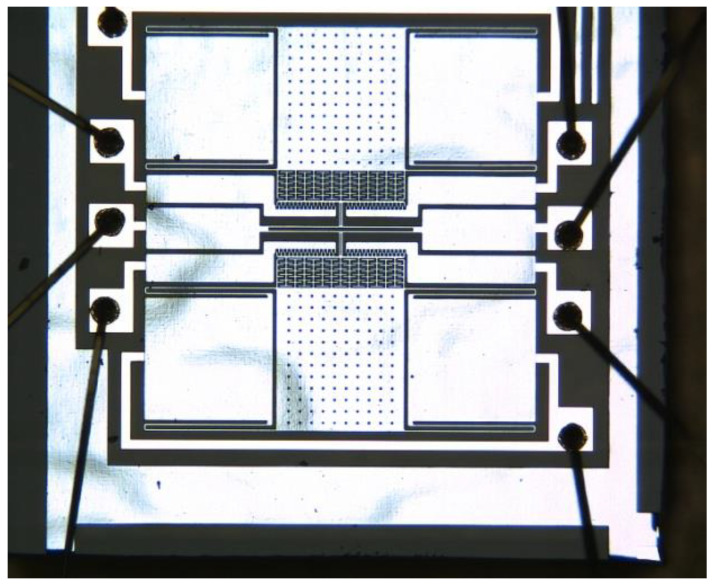
Fabricated microstructure (single-stage beam).

**Figure 11:**
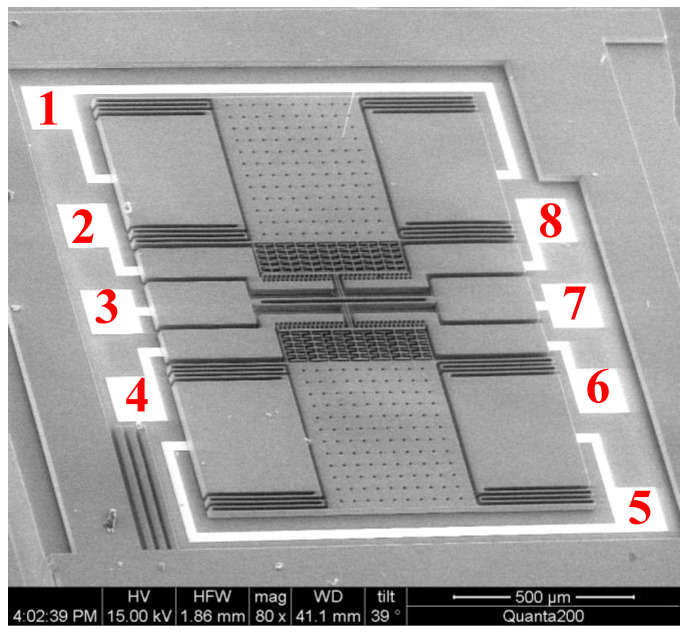
Fabricated microstructure (double-stage beam).

The structure layer was composed of monocrystalline silicon material doped with concentrated boron to improve the conductivity of the microstructure [13]. The substrate was pyrex 7740 glass, and the anode bonded the microstructure and the substrate [7]. For larger depth and width ratios, three photolithography analyses were conducted with an inductive coupled plasma (ICP) emission spectrometer) [14]. Under the condition ofthe etching depth being 40 μm, the metal electrodes were welded after tape-out. The fabricated single-stage and double-stage folded beam accelerometers are shown in Figure 10 and Figure 11. The structure was complete and there was no microstructure fracturing orshort-circuiting. The electrode layer had eight electrodes, as shown in Figure 11, of which the third and seventh electrodes connected to the tuning fork beam were short-circuited. There weretwo groups of driving combs on the upper and lower sides; each group of combs had two electrodes. The second and eighth electrodes were short-circuited and connected with the driving voltage, the same was true of the fourth and sixth electrodes, and the same was true of the first and fifth electrodes.

**Figure 12:**
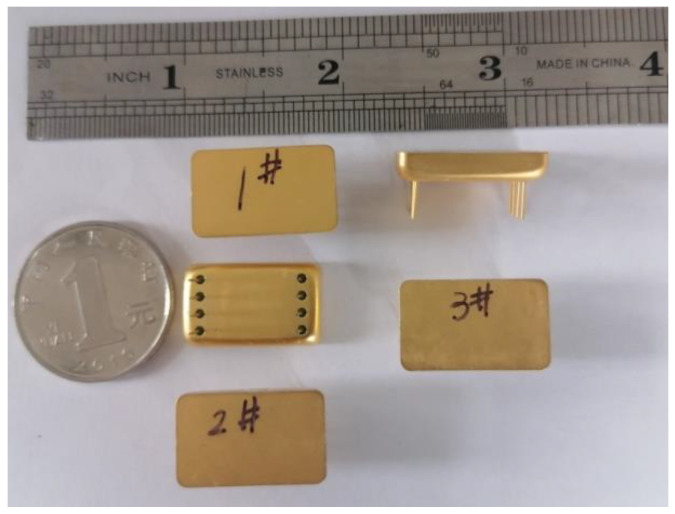
Metal encapsulated sensor.

The estimated quality factor of the microstructure was less than 100 under standard atmospheric pressure packaging. When the AC and DC voltages were both less than 5 V, the vibration amplitude was small, the detection capacitance changed slightly, and the interface circuit was difficult to detect. To reduce the resonance energy consumption, the core structure was vacuum-encapsulated in a metal tube and shell (20–30 mTorr), and the packaged accelerometer was as shown in Figure 12.

**Figure 13:**
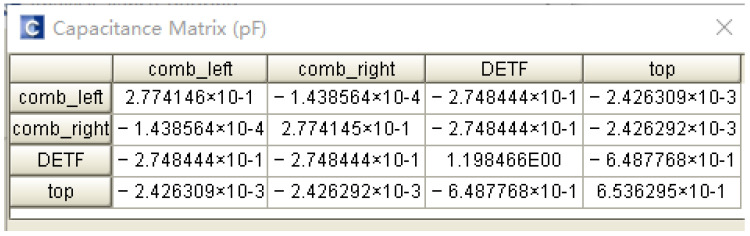
Interface capacitance simulation.

In selecting driving and detection voltages, we needed to estimate the value of the driving and detection capacitances first, before the driving and detection voltages were finally converted into electrostatic forces through the capacitances. The entire microstructure was symmetrically divided into two equal parts, the top and bottom. The CoventorWare software was used to establish the physical simulation model of the detection capacitance and the driving capacitance. Differing from the previous modal simulation and force analysis, the mesh setting for the microstructure must be performed after the 3D modeling of the equivalent micromechanical resonant accelerometer was completed. The electrostatic field’s finite element analysis was selected, the relevant constraints were set on the microstructure, and the capacitance matrix was finally obtained [17]. The finite element simulation results show that the comb capacitance of the left and right parts of the fixed driving comb and the tuning fork beam was 0.2748 pF; that is, the two driving capacitances were both 0.2748 pF. The detection capacitance formed by the flat plate on the proof mass and the flat plate on the tuning fork beam was 0.6488 pF, and the coupling capacitance between the fixed driving comb and detection plate was 0.002426 pF. The simulation results are shown in Figure 13.

**Figure 14:**
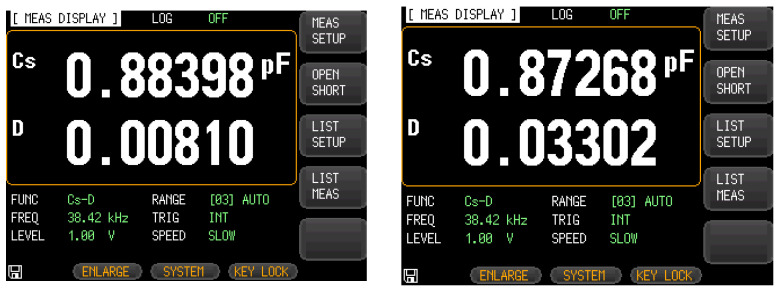
Interface capacitance test (driving capacitance).

**Figure 15:**
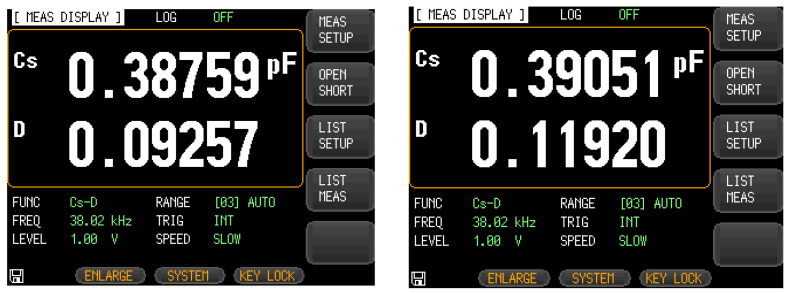
Interface capacitance test (sensing capacitance).

The probe of the LCR measuring instrument was used to clamp the corresponding pins after packaging and adjust the measurement excitation frequency. Figure 14 shows that the driving capacitance values of the single-stage and double-stage folded beam accelerometers were 0.88398 pF and 0.87268 pF, and the corresponding detection capacitances were 0.38759 pF and 0.39051 pF, respectively, as shown in Figure 15. Considering the fixture and welding of metal leads and the process manufacturing errors, the test results and simulations were found to be of the same order of magnitude and basically consistent.

**Figure 16:**
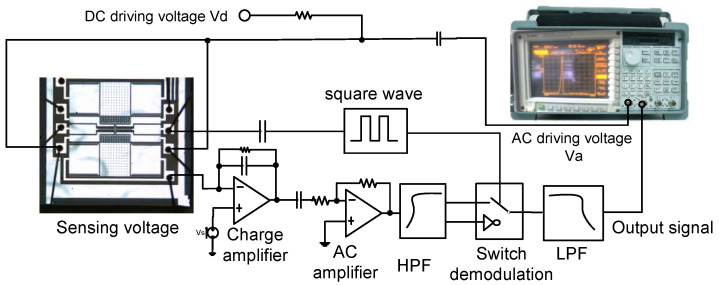
Open-loop test circuit.

To obtain the quality factor and resonant frequency, an Agilent 35670A dynamic signal analyzer was used for the open-loop frequency sweep test. The third and seventh electrodes were connected to an 866 kHz square wave, and the fourth and sixth electrodes were both connected to DC and AC drive voltages. The DC-regulated power supply provided the DC voltage, and the AC voltage was supplied by the output port of the dynamic signal analyzer. The detection voltage is provided by the manual programmable potentiometer divider and was connected to the positive input terminal of the charge amplifier of the interface circuit. The detection capacitor was output through the charge amplifier, DC blocking amplifier, high-pass filter, switch demodulation module, and low-pass filter, and then connected to the input of the dynamic signal analyzer. The circuit schematic is shown in Figure 16.

**Figure 17:**
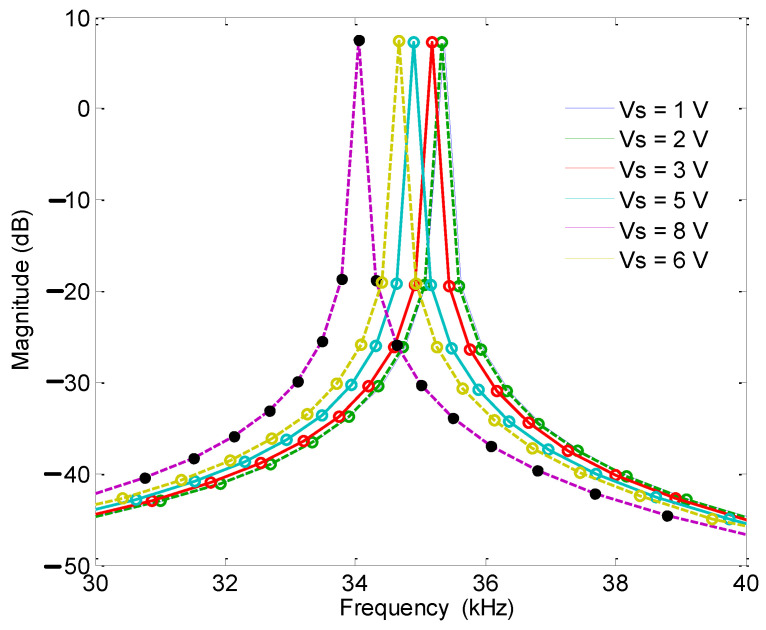
Amplitude frequency curve (double-stage beam).

**Figure 18:**
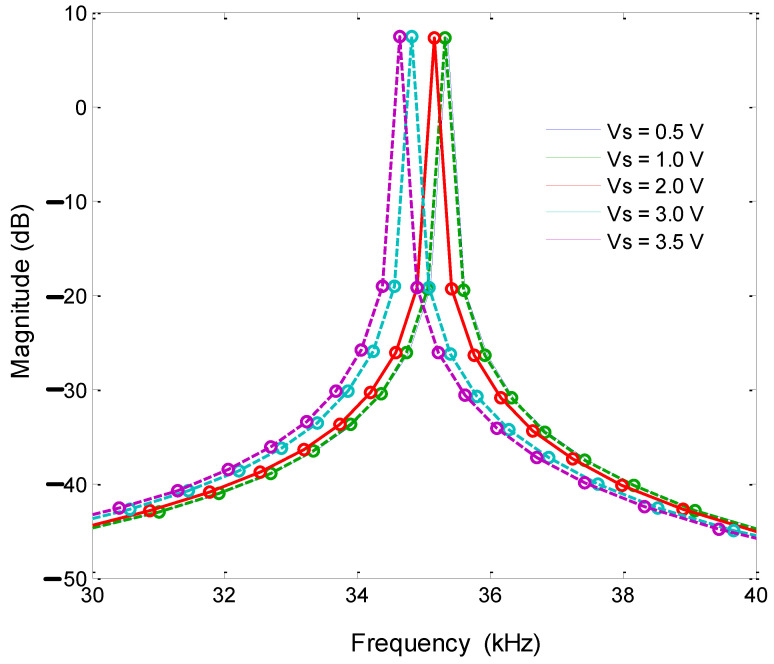
Amplitude frequency curve (single-stage beam).

Under the condition of the accelerometer being placed flat (the acceleration in the X-axis and Y-axis directions was 0), the frequency sweep range was 30–40 kHz, the DC driving voltage was 1 V, and the AC voltage amplitude was 2 V. A resistor was used to adjust the detection voltage and a general-purpose interface bus (GPIB) cable was used. The test amplitude–frequency characteristic curve showed that both the single-stage and double-stage folded beam accelerometers could change the resonant frequency by changing the detection voltage. However, the adjustment of the double-stage folded beam was more sensitive. The frequency corresponding to the double-stage folded beam changed greatly with the same detection voltage, as shown in Figure 17 and Figure 18. At the same time, the two resonant beams of the same accelerometer were tested, and they showed that the amplitude–frequency curve was asymmetric concerning the center resonant frequency, and that the resonant beam had nonlinear characteristics when the electrostatic force was large.

**Figure 19:**
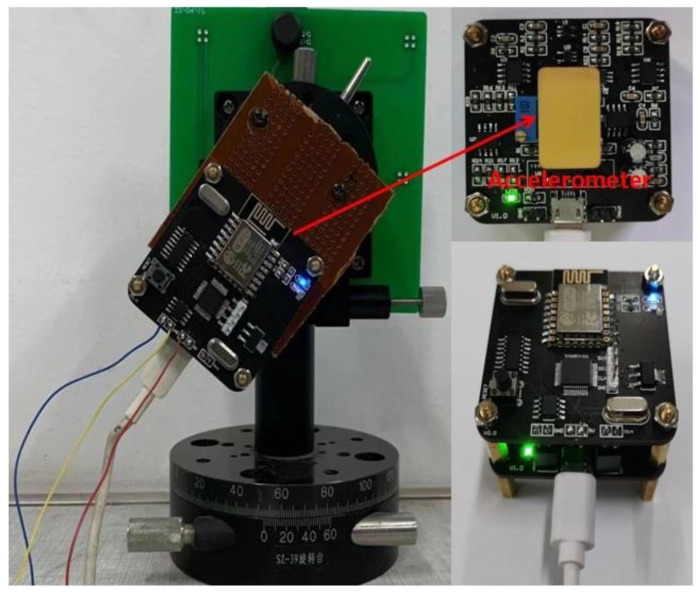
Experimental circuit.

The self-made rotary slide and dial could achieve a precise adjustment of 0.5°, providing acceleration input from −1 g to 1 g through gravity decomposition. The test instrument is shown in Figure 19, and the corresponding voltage supply mode remained unchanged.

**Figure 20:**
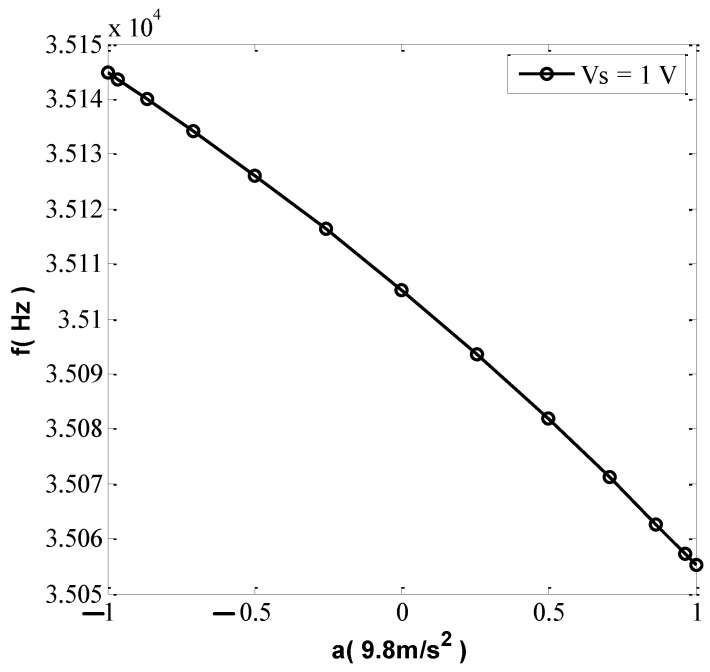
Output characteristic test (Vs = 1 V).

**Figure 21:**
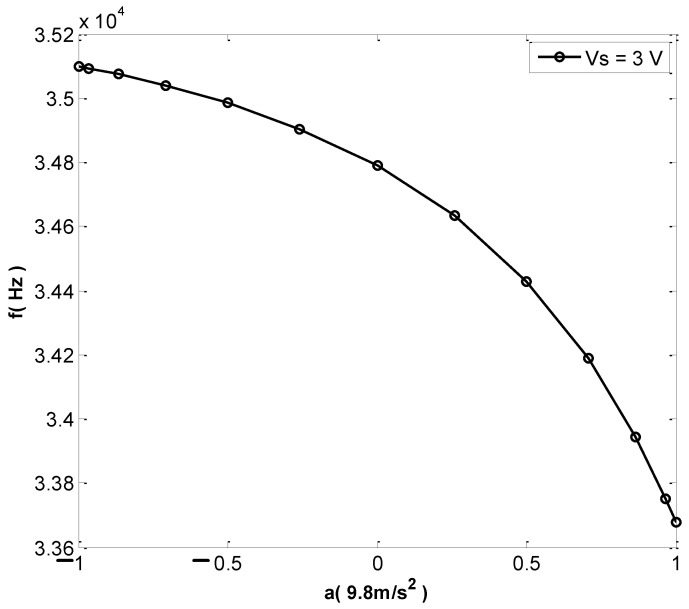
Output characteristic test (Vs = 3 V).

The encoder was adjusted to achieve an angle difference of 15° of rotation and decompose the acceleration into the corresponding sine components. When the detection voltage changed to 1 V and the corresponding acceleration rotation angle was from −90° to 90°, the relationship between acceleration and resonant frequency was as shown in Figure 20, and the sensitivity was 44.5 Hz/g. When the detection voltage was 3 V, the relationship between acceleration and resonant frequency was as shown in Figure 21. A large detection voltage corresponds to a high sensitivity, but the resonant frequency and acceleration have a severely nonlinear relationship.

**Figure 22:**
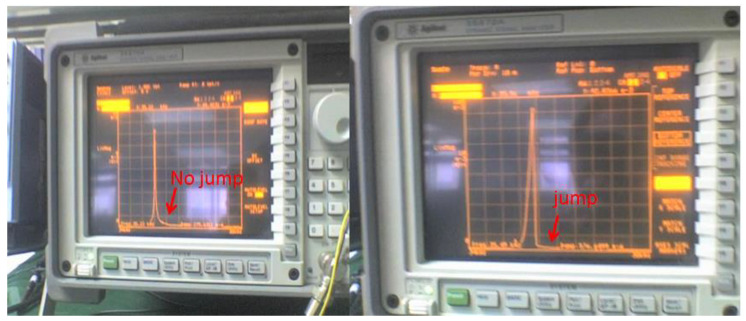
Nonlinear characteristics of the accelerometer.

The amplitude of the AC drive voltage was kept unchanged, and the DC stabilized power supply was adjusted to change the DC drive voltage. When the DC detection voltage is 3 V, the amplitude–frequency curve does not jump; when the DC detection voltage is 5 V, there is a jump in the amplitude–frequency curve. See Figure 22 for the curve. The larger the DC driving voltage was, the more the resonant frequency was inclined toward the direction of frequency increase. The analysis showed that the microstructure featured a nonlinear coupling of amplitude and frequency when the driving voltage and detection voltage were large [15].

**Figure 23:**
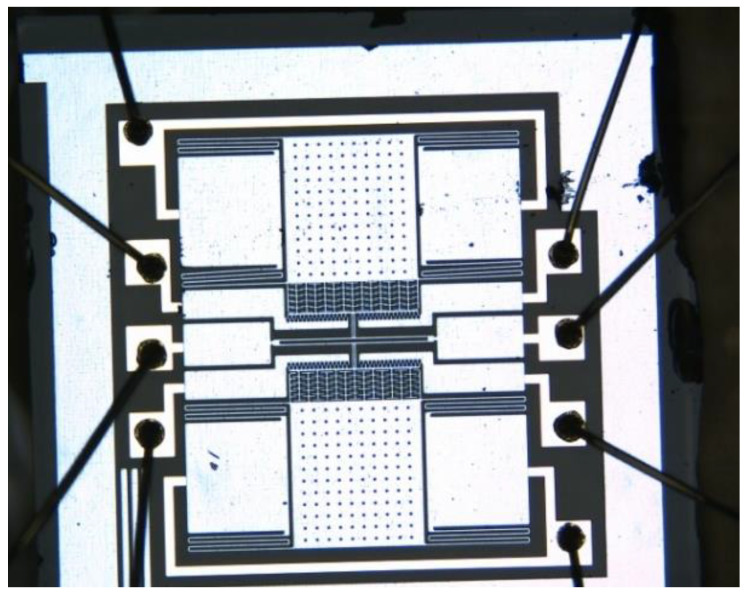
Deformation of double-stage folded beam.

**Figure 24:**
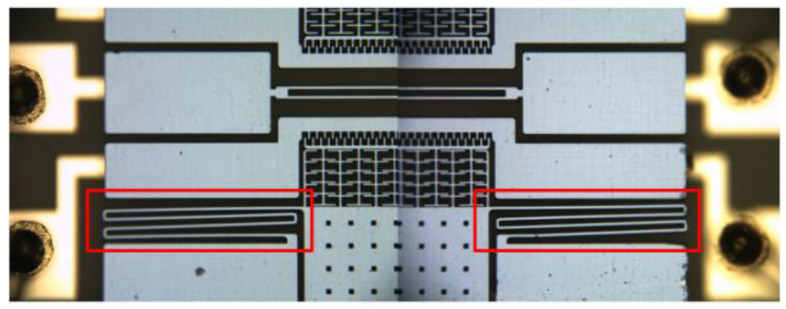
Deformation of double-stage folded beam (zoomed in).

The microstructure was observed with a microscope. Compared with the situation shown in Figure 11, the double-stage folded beam was irreversibly deformed, as shown in Figure 23; this is partially enlarged in Figure 24. Obviously, if the detection and driving voltages are increased, the double-stage folded beam will be prone to failure, which affects its performance. This aspect should be considered when designing the structure layout of an accelerometer.

**Figure 25:**
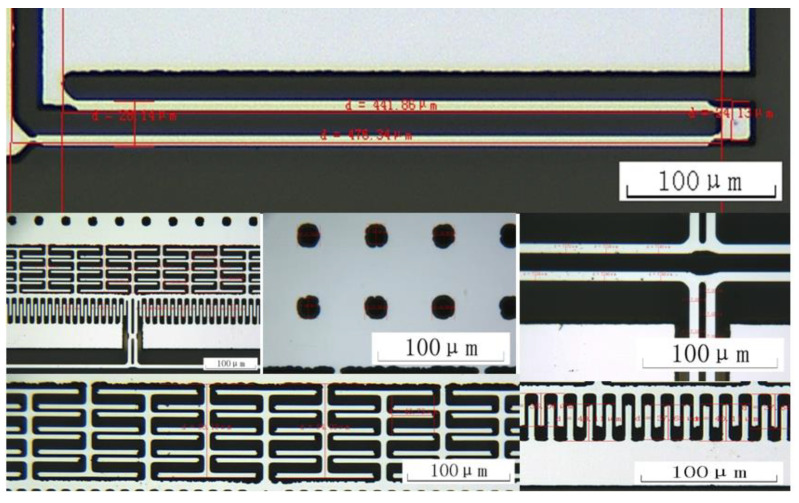
Computer vision measurement of the microstructure size.

**Figure 26:**
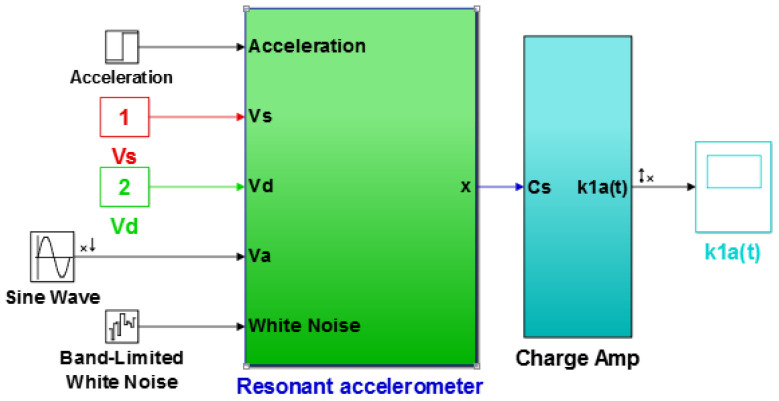
Numerical simulation model of the accelerometer.

**Figure 27:**
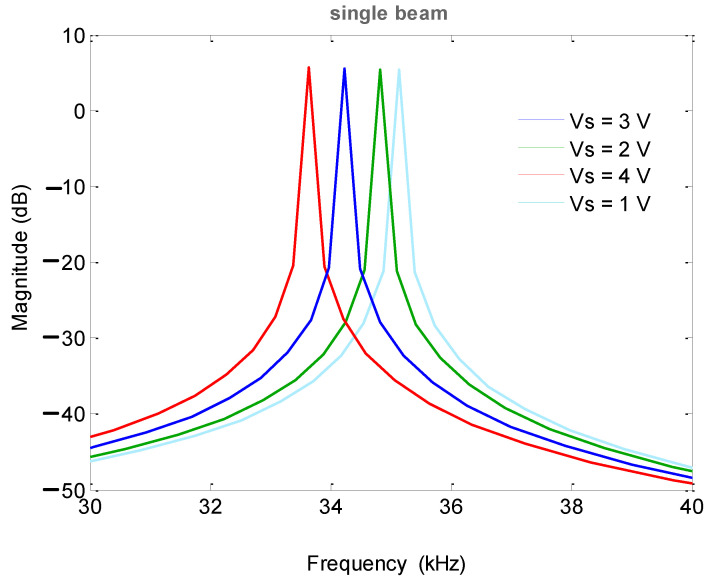
Amplitude-frequency curve of the simulation model.

Before microstructure encapsulation, the computer vision method was used to directly mark the length and width dimensions, as shown in Figure 25. Table 3 shows the structural parameters designed and measured. Combined with the quality factor of the experimental test, the numerical model of the accelerometer (including the interface charge amplifier) was obtained, and the numerical model was established under Matlab/Simulink. The external inputs were acceleration, detection voltage, DC driving voltage, AC driving voltage, and white noise, and the output terminal was the charge amplifier, as shown in Figure 26. The linear analysis tool was used to analyze the amplitude–frequency characteristic curves under different detection voltages, as shown in Figure 27. At 30–40 kHz, the resonant frequency was decreased by changing the detection voltage from 1 V to 4 V. The amplitude–frequency curve was asymmetrical, but the simulated vibration amplitude was slightly smaller than that of the experiment, and the detection voltage corresponding to the frequency reduction was inconsistent. The simulation error was related to the circuit parameters of the interface. As the interface circuit focuses on the frequency measurement [18,19,20], the effect of the error under small amplitude on the frequency measurement can be ignored.

## 5. Conclusions

The present work concerns the operating principles and a thorough experimental characterization of a new polysilicon resonant micro accelerometer based on electrostatic stiffness. The relationship between the stiffness of the folded beam and the stiffness of the resonant beam has to be constrained in order to better realize the controllable design of the sensitivity. When the stiffness of the folded beam is much lower than that of the resonant beam, the influence of the displacement of the resonant beam on the sensitivity can be ignored. The open-loop test was used to validate the relationship between the resonant frequency and the detection voltage, which verified the positive effect of the frequency adjustment of the electrostatic negative stiffness. At the same time, the amplitude–frequency curve was not point-symmetric about the resonant frequency, and a large driving voltage caused a nonlinear coupling of the amplitude and frequency. The large detection voltage also caused the failure of the double-stage folded beam, and the subsequent measurement and control circuit needed to maintain the small-amplitude resonance of the MEMS resonant sensor.

## Figures and Tables

**Table 1:** Static force analysis (single-stage beam).

Acceleration (m/s^2^)	Displacement (um)	Stiffness (N/m)
2	0.00773	9.4398
4	0.01546	9.4398
6	0.02319	9.4398
8	0.03092	9.4398
9.8	0.03788	9.4398

**Table 2:** Static force analysis (double-stage beam).

Acceleration (m/s^2^)	Displacement (um)	Stiffness (N/m)
2	0.01427	5.1127
4	0.028544	5.1127
6	0.04282	5.1127
8	0.05709	5.1127
9.8	0.06994	5.1127

**Table 3:** Structure parameters of the accelerometer.

Parameters	Units	Design	Measurement
Length of fold beam	μm	500	479
Width of fold beam	μm	8	7.01
Spacing of fold beam	μm	14	16.1
Length of connecting beam	μm	160	149.2
Width of connecting beam	μm	9	7.6
Spacing of connecting beam	μm	4	4.93
Length of drive comb	μm	40	38.5
Width of drive comb	μm	5	4.78
Spacing of drive comb	μm	2	2.5
Pairs of drive comb	pair	19	19
Length of DETF	μm	700	662.4
Width of DETF	μm	8	7.4
Length of detect plate capacitor	μm	50	45.3
Width of detect plate capacitor	μm	6	4.88
Spacing of detect capacitor 1	μm	2	2.46
Spacing of detect capacitor 2	μm	10	10.56
Total pairs of detect capacitor		40	40
Length of proof mass	μm	620	609
Width of proof mass	μm	700	684
Length of damping hole	μm	10	12.38
Width of damping hole	μm	10	12.85
Number of damping hole		110	110
Structure layer thickness	μm	40	40.3

## Data Availability

All data are true and reliable.
